# Potential design problems for ITER fusion device

**DOI:** 10.1038/s41598-021-81510-2

**Published:** 2021-01-22

**Authors:** A. Hassanein, V. Sizyuk

**Affiliations:** Center for Materials Under Extreme Environment (CMUXE), Purdue University, West Lafayette, IN 47907 USA

**Keywords:** Energy science and technology, Engineering, Materials science, Mathematics and computing, Optics and photonics, Physics, Plasma physics

## Abstract

The international thermonuclear experimental reactor (ITER) is a worldwide project currently being built in France for the demonstration of the feasibility of thermonuclear technologies for future realization of successful commercial fusion energy. ITER is of the tokamak based design using strong magnetic fields to confine the very hot plasma needed to induce the fusion reaction. Tokamak devices are currently the front leading designs. Building a successful magnetic fusion device for energy production is of great challenge. A key obstacle to such design is the performance during abnormal events including plasma disruptions and so-called edge-localized modes (ELMs). In these events, a massive and sudden release of energy occurs quickly, due to loss of full or partial plasma confinement, leading to very high transient power loads on the reactor surface boundaries. A successful reactor design should tolerate several of these transient events without serious damages such as melting and vaporization of the structure. This paper highlights, through comprehensive state-of-the-art computer simulation of the entire ITER interior design during such transient events, e.g., ELMs occurring at normal operation and disruptions during abnormal operation, potential serious problems with current plasma facing components (PFCs) design. The HEIGHTS computer package is used in these simulations. The ITER reactor design was simulated in full and exact 3D geometry including all known relevant physical processes involved during these transient events. The current ITER divertor design may not work properly and may requires significant modifications or new innovative design to prevent serious damage and to ensure successful operation.

## Introduction

The International Thermonuclear Experimental Reactor (ITER) is currently being built in France to produce, for the first time, a fusion power of about 500 megawatts thermal output power, i.e., a gain of ten times more than the input power of about 50 megawatts of thermal power needed to demonstrate the fact of producing more thermal power from the fusion process than is used to heat the plasma. ITER is not designed to produce sufficient power for electricity consumption. The project is international including the European Union as one entity and six other countries of India, Japan, China, Russia, South Korea, and USA.

ITER design is based on the tokamak concept which uses strong magnetic fields to confine the hot plasma to induce thermonuclear reaction and as a result produce power. The stored thermal and magnetic energy in the hot plasma can lead to plasma instabilities and loss of plasma confinement, i.e., plasma disruptions^1^. Loss of plasma confinement is a serious issue in tokamak devices that threatens successful operation of the tokamak designs. During ITER and future tokamaks operation, many physical processes take place in an integrated self-consistent matter, which cannot be fully studied experimentally in current tokamak devices due to the large power density anticipated in future devices. Logically, there are two ways to predict future operation of such devices: (1) to extrapolate current experimental data taken from the existing much smaller tokamaks^2^; and/or (2) to simulate various models involved where all important physical processes in real three dimensional (3D) device geometry should be integrated self-consistently, i.e., with an end-to-end or all-inclusive model^3^.

Plasma instabilities are of major concern in advancing magnetic fusion for reliable energy production. The anticipated increased in electromagnetic loading and the heat fluxes to plasma facing components (PFCs) during these instabilities will result in serious components damage. The high confinement regime (H-mode) is currently the reference scenario for ITER device. At the same time after the transition from the low confinement (L-mode) regime to the H-mode, new plasma oscillations are activated at the plasma border, so-called Edge Localized Modes (ELMs). The theoretical calculations predict the ELM periodical generation and decay or about ~ 1 Hz. During ELMs, the hot hydrogen isotope plasma loses part of its energy in the form of energetic particles escaping confinement and cause significant surfaces heating of PFCs. The ELMs are a consequence of the additional plasma heating as a result of the increased confinement time.

As the power and size of fusion devices increase they face new obstacles and challenges. A key obstacle to the successful energy production in tokamak reactors is plasma material interactions and robust performance of PFCs during abnormal events including ELMs at normal operation and disruptions during abnormal operation. In a disruption the entire plasma energy is lost at once due to such magnetohydrodynamic (MHD) instabilities and about 10% of the plasma energy is lost due to ELMs event occurring at normal operation. While significant efforts are being pursued to find reliable and robust ways to predict and mitigate such events^1^, a credible design must withstand a few of these transient events, that will take place during operations, without significant damage. Most of the particles escaping from the core plasma is gyrating along the magnetic field lines until reaching the strike point (SP) area on the divertor plate. To prevent severe damage of the PFCs, the "wetting area" on the divertor plate should be enlarged to decrease the surface heat load. Generally, there are two possible ways to increase the wetting area and reduce the heat load: (i) by optimization of the divertor magnetic configuration and components design; or (ii) by establishing a buffer zone between the escaped core plasma and SP to dissipate the heat load in current tokamak designs.

The divertor detached regime belongs to the second way and consists in timely injection of neutrals into the divertor space^4^. The neutrals will be ionized by the core escaped particles and dissipate the energy by photon reradiation above the PFC surface. Therefore, the "wetting area" will be enlarged. The preferred use of low-Z materials to act as buffer zone requires higher concentration of the injected material to ensure sufficient energy dissipation that could further affect the core stability. The lower amount of higher-Z seeding could also affect the confined plasma, which may result in considerable upstream temperature drop^5^. The detached divertor can mitigate relatively small ELMs from the impurity radiation localized in the cold region of the divertor^4^. However, the large ELMs burn through the detached divertor plasma and can seriously damage the divertor plate and indirectly damage the nearby components. The optimum solution of such serious problem is in the correct optimization of the divertor design, magnetic configuration, and detachment/mitigation methods. Successful design of fusion reactors will critically depend on the accurate prediction of all the heat and particle loads incident on various interior reactor components to correctly identify the optimum materials for PFCs. The loss of core plasma particles confinement are a serious concern to the lifetime of the divertor plate and surrounding components and a potential source for immediate and subsequent core plasma contamination from the eroded/splashed materials from damaged components.

This work carefully predicts and simulates the evolution and propagation of the secondary plasma generated from the divertor plate material, its interactions with various reactor components following a disruption or an ELM event initially incident on both the inner and outer divertor plates. This secondary divertor plasma has completely different physics and hydrodynamic evolution compared to the core DT plasma^6^. While disruptions and ELMs are much different in energy content, they both have serious consequences to the performance of the current divertor designs in the tokamak concept for ITER and beyond.

## Challenges of finding suitable materials for plasma-facing components

The escaping core plasma particles will follow the magnetic field lines and interacts with the divertor plate designed to handle and tolerate high heat and particle loads. In the past, carbon-based materials were proposed as divertor plates since carbon doesn’t melt. However, carbon has several disadvantages including significant neutron degradation, retention of tritium, higher chemical and sputtering erosion, etc. Tungsten was then proposed to replace carbon as the main divertor material about two decades ago. While tungsten has better capabilities in handling high heat flux, lower sputtering erosion, and more resistant to neutron damage being a metallic material, it also has few disadvantages including morphological changes during plasma interactions and high radiation power being a high-Z material. Nevertheless, during the early phase of ELMs and disruptions the escaped plasma particles interact with the divertor plate material, heating it to high temperature and form a cloud of vapor that shields and protects the divertor plate from further direct interaction with the incoming escaping plasma particles^7^. The remaining incoming plasma particles will then interact with this vapor cloud ionizing it and form a secondary plasma. The continuous interaction of the disrupting plasma particles dynamically with the developed and evolving secondary divertor plasma will ultimately determine the potential damage and lifetime of all interior reactor components.

We have recently enhanced our three-dimensional models for the comprehensive integrated simulation of core plasma deposition and interaction with the divertor and surrounding components during loss of confinement^8^. Initially, the disrupting core plasma initiates the flow of particles in the actual three-dimensional ITER geometry within the presence of both strong magnetic and electric fields beginning from the last closed magnetic flux surface into the scrape-off-layer (SOL) area and up to the penetration depths within the divertor components. The simulations initially used the equilibrium configuration of the magnetic field provided from the database of the EQDSK files^9,10^. We have used various numerical methods in our HEIGHTS simulation package. These methods include new three-dimensional Monte Carlo (MC) kinetic model to simulate the spatial distribution of ion and electron energy depositions of the incident core particles on the inner and outer ITER divertor plates. These advanced numerical methods were extensively verified and benchmarked in current tokamak devices^6,8^. The simulations also include detail implementation of the complex magnetic field structure and the exact geometry of various ITER internal components. The secondary plasma models, characteristics, and conditions, are completely different than the core DT plasma conditions. The conditions expected in the secondary plasma allowed us to use our detail comprehensive models developed in HEIGHTS-EUV package to simulate current plasma devices for non-fusion nanolithography applications^11^. The magnetohydrodynamic, atomic and plasma physics, plasma opacity and emissivity, photon radiation transport, and plasma heat conduction processes can be simulated in such dense secondary plasma similar to those generated in laser- and discharge-produced plasma devices currently proposed for the advanced extreme ultraviolet (EUV) nanolithography. The dense generated laser and discharge plasmas will emit radiation fluxes that are similar in magnitude to the incident core plasma particles^8,12^. Our developed gyrokinetic model of the escaping core plasma and its deposition is used to simulate the dynamic evolution of the divertor secondary plasma. The simulation follows from the initiation at divertor surfaces, propagation along components surfaces, and its penetration into the SOL. These integrated models include heating and phase change of component surfaces, erosion, melting, ablation, ionization, mini plasma formation, secondary plasma magnetohydrodynamic evolution, heat conduction in plasma, and photon radiation generation and transport. The magnetohydrodynamic equations are applied here to the secondary plasma evolved from the divertor plate and not to the hot DT rarefied core fusion plasma where the particles of the escaped core plasma provides the energy source feeding this dense plasma. The density of this divertor generated plasma is much higher than that of the hot fusion plasma and are not be fully ionized as the core fusion plasma.

## The need for integrated simulation

Because future tokamak devices like ITER are much larger than current devices it is hard to predict performance based on available data from current machines. Therefore, one must rely on models and computer simulation to predict performance of future devices. The modeling efforts of plasma behavior in magnetic fusion are usually separated into two general areas: modeling of burning and confinement of the pure DT fuel plasma, and modeling the response of PFCs to steady state and transient particles and heat loads escaping confinement. These two main areas were never overlapped or integrated together due to the small sizes of current tokamak devices, the very short plasma duration, and the low power density in these devices. It was also assumed that the internal plasma facing and nearby surfaces, other than the divertor plate, will not have any significant heat loads due to ELMs and disruptions as a result of the divertor response to such impact.

When the tokamak power is increased to cause significant erosion of the divertor plates during transient events at normal and abnormal operations, the plates will melt and vaporize the divertor material soon after the start of the transient events. Further heating from the escaping plasma particles will continue to heat the vaporized material and form a secondary divertor plasma that expands following the magnetic field lines into the scrape-off layer and continue to interacts with the incoming DT plasma^6^. Assuming the damage from the transient events to only affect the divertor plate which claimed to be easily repaired or replaced is no longer the case due to the involvement of the secondary plasma formed from vaporized divertor materials^13^. This secondary divertor plasma generation, evolution, and its dynamic interaction with the disrupting DT plasma will determine the overall damage to all interior components during such events. Therefore, the earlier discussion and assessment regarding various potential divertor materials, i.e., carbon, tungsten, and liquid metals didn’t take into account the consequences of their response to transients and their resulting plasma evolution on the overall damage and lifetime.

While tungsten is very resistant to erosion as a plasma-facing material however, being a high-Z material it is a strong radiative material as a secondary generated plasma or if entered into the core plasma. The low-Z carbon material is a weak radiator but has lower resistance to neutron damage and potential high tritium inventory. Liquid metals, while can overcome several of these issues, may be harder to implement in reactors, particularly in the current ITER device, and could also cause more damage in other places when considering their integrated response to transient events. Preliminary estimations (both theoretical and experimental) can accurately estimate ITER power and particle fluxes on the divertor plate surfaces during transients and loss of confinement^14,15^. The incident power/particle loads determine the response of divertor materials, its evolution, vaporization, and its plasma formation and expansion into the divertor space and SOL. The intensity of divertor vaporization and secondary plasma generation and its characteristics depends on the value of the power flux at the SP. The estimated values of the power flux are determined by various methods from the nuclear fusion reaction processes. Good agreement exists in the literature for the values of total energy of disruption thermal quench, i.e., 120–175 MJ and about 10% of this value is estimated for ELMs, i.e., up to 17.5 MJ^15,16^.

**Figure 1:**
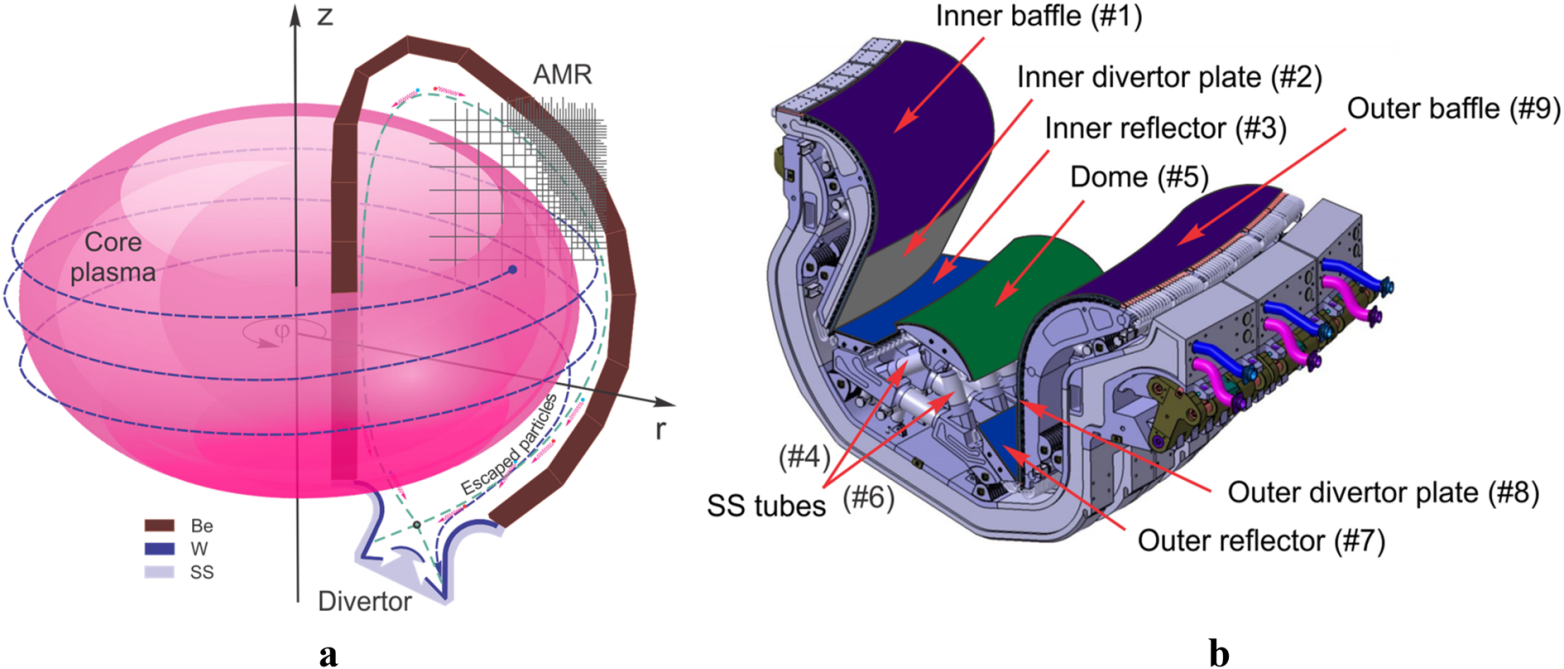
3D schematic illustration of ITER components and coordinate system (**a**), and divertor and nearby component surfaces (**b**) implemented in HEIGHTS package. The images were prepared using CorelDRAW Graphics Suite 11.

Reliable computer simulations and models are needed for accurate predictions of current and future fusion devices. Simulation of such events requires integrating various interaction processes including particle and photon energy deposition, material thermal evolution, phase transformation, melting, vaporization, atomic physics models, ionization, plasma formation, MHD evolution, plasma opacity, and photon transport in strong magnetic and electric fields environment and in full 3D real reactor design. Recently, we significantly enhanced our comprehensive HEIGHTS simulation package to efficiently integrate various models of different interaction processes implemented for the exact current ITER design^6^. Figure 1 illustrates the simulation domain of ITER internal plasma facing components using the adaptive mesh refinement (AMR) to map details of the design from submicron to tens of meters scale. In our modeling, we integrated two approaches: kinetic Monte Carlo approach for the description of the hot rarefied hydrogen plasma and the MHD approach for the secondary divertor plasma evolution. The dense generated divertor plasma is treated as a fluid and the energy exchange between core plasma and secondary plasma is implemented as collisions among individual particles. The solution of the MHD equations then determines the changes in magnetic fields structure and the secondary plasma propagation dynamics. The changes in magnetic fields configuration controls the MC particle motion and trajectories.

The detailed simulation of the core particles motion into the SOL (Fig. 1a) is used in our comprehensive model as an input power source to activate the magnetohydrodynamic models in HEIGHTS package^6^. Each time step simulation requires at least ~ 10^3^ particles to achieve reasonable compromise between accuracy of calculation and simulation time. The MHD time step taken is 50 ps, for an ELM duration of 1 ms. As a result, 2 × 10^7^ MHD time steps are used during the ELM duration of 1 ms. The MC ELM module is invoked every 100 MHD time step. Therefore, the total number of particles used is 2 × 10^5^ × 1000 = 2 × 10^8^ particles for the full simulation of the ELM event. We recalculate plasma properties every hydrodynamic time step in each cell and also the photon radiation transport (RT) in the dynamically propagating secondary plasma and generate updated opacity data regarding the absorption and emission processes^17^. The time-dependent details of the absorbed photons in various plasma facing surfaces and nearby components predict the time dependent energy deposition on all interior tokamak components during ELMs and disruptions. Photon radiation transport in the evolving divertor plasma is critical in evaluating various damage to interior components. Photons are not affected by the magnetic field and are free to move and deposit their energy to any component with direct line of sights. Accurate radiation transport calculations are important in assessing the damage to these components. These calculations are very time consuming and require innovative solution methods and supercomputer clusters to simulate ITER machine real geometry in a reasonable time with good accuracy. We have developed both extensive Monte Carlo and ray tracing models^6^ where we included detailed 3D photon transport within the secondary plasma of the tungsten material as chosen for ITER divertor plates. We numerated the divertor cassettes surfaces as shown in Fig. 1b. Our new optimized Monte Carlo method significantly reduced the solution time by more than two orders of magnitude compared to the ray tracing methods within the same needed accuracy^18^. The Monte Carlo radiation transport calculations were performed for full photon spectrum (from 0.05 to 10^5^ eV) in each hydrodynamic cell with selection of ~ 2 × 10^4^ optical groups for the tungsten plasma. Every time-step radiation transport calculation used up to 10^4^ quasi-photons per hydrodynamic cell depending on the cell weight factors^8^. Further increase in the number of hydrodynamic domain cells, number of particles and photons in each cell, details of photons spectra, and number of optical opacity groups will further increase the accuracy of such simulation. The values chosen here were optimized and carefully checked for this study and are sufficient for accurate assessments in reasonable simulation time.

The dynamic Monte Carlo simulation of the incident DT plasma particles is integrated with various particle interactions and plasma collisional processes of this evolving secondary plasma; starting from the core borders and ending in the PFC walls. Implementing details of the collisional/scattering processes and proper interaction cross sections provide the needed details of energy deposition into the SOL plasma and the scattered particle showers deposition and penetration into various plasma-facing and surrounding internal components. Initially, the SOL is near vacuum/low gas density. The collisional/scattering probabilities of the D, T, and e in SOL are therefore small. The disrupting/escaped core plasma particles start randomly along the entire last closed flux surface (LCFS). The initial direction of the particles was sampled as 3D equiprobable so that electrons and ions can either be returned to the core plasma or can move along the "passed" or "trapped" trajectory^8^. Then the solution of the heat conduction equation as a result of photons and particles deposition in each internal component determines the thermal response, melting depth, and vaporization erosion in all plasma-facing and nearby surface components.

**Figure 2:**
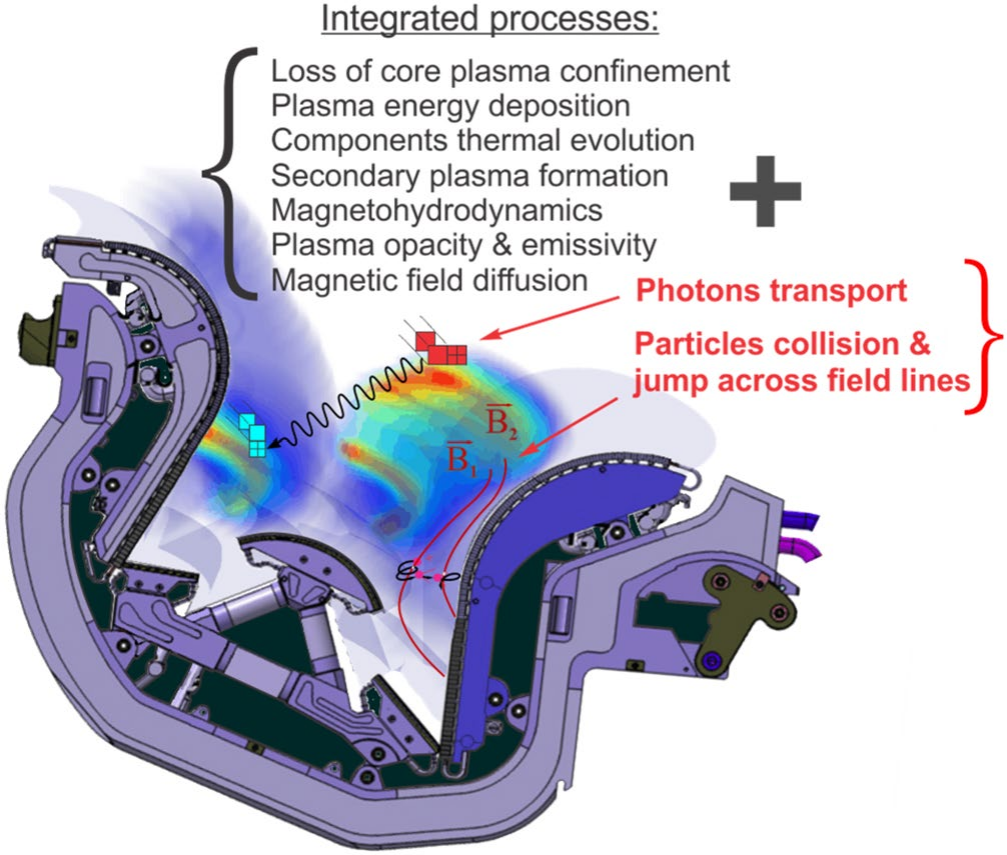
Integrated physical processes implemented in HEIGHTS. The images were prepared using CorelDRAW Graphics Suite 11.

The vaporization of tungsten divertor material either in the typical expected case of 1.0 ms disruption (complete loss of confinement) or ELM (during normal operation), is sufficiently large and as a result the divertor space will quickly be occupied with tungsten vapor/plasma with a density of several orders of magnitude higher than the original incident DT fuel plasma. The divertor plate will then be fully shielded from direct interaction with the incoming DT plasma early in time during ELMs and disruptions. The incoming core plasma particles will then deposit its energy into the tungsten vapor cloud and can be scattered to various locations from the front of the evolving dense divertor vapor cloud. The escaped particles determine the initial energy source and boundary conditions needed for the MHD evolution of the divertor generated plasma^6^. The energy deposited into the evolving vapor will result in further heating and generation of a tungsten secondary plasma. The divertor-generated secondary plasma evolution and propagation again is the main determining factor in the resulting damage to all plasma-facing and hidden components which has been overlooked in all past studies including the ITER team. Figure 2 shows the integrated physical processes implemented in HEIGHTS to accurately simulate the response of various components to transient events.

While the total energy released in an ELM during normal operation is only about 10% of the total energy stored in the core plasma and for ITER it is about 12.6 MJ, the evolving dense secondary plasma is strongly resistant to the motion of the incoming DT plasma flow due to the large difference in plasma density. Our simulations showed a tungsten plasma density around ~ 10^17^ cm^−3^ is developed near the original strike divertor surface that is much higher than the DT core plasma value ~ 10^13^–10^14^ cm^−3^. The DT core plasma mass transport and penetration into the secondary divertor plasma is negligibly small. The scattered core plasma particles transfer part of their kinetic energy in the evolving secondary plasma, therefore influencing the dynamics evolution of this divertor secondary plasma as well as deposit their remaining energy as particle showers into various nearby components causing further potential damage. Consequently, the dense dynamic secondary plasma sweeps and moves the frozen magnetic field lines and therefore, additionally changes the disrupting DT core plasma particles flow and further redirect scattered flux showers into other components.

Contrary to the pure hydrogen isotope high-temperature plasma (fuel plasma), the low-temperature dense high-Z secondary plasma has much higher radiation power. In addition to the complex MHD of the secondary plasma, the fine details of plasma opacity and photon radiation transport processes must be included and implemented into the full 3D exact reactor design. During the transient events, the hydrogenic plasma particles transfer their energy into the developed secondary plasma by various collisional processes. The secondary plasma is then heated, ionized, excited, and as a result intensively reradiates photons. Usually, photon radiation transport of the secondary plasma is not included into most tokamak simulation codes^19,20^. The evolved tungsten secondary plasma from both inner and outer divertor plates absorbs the majority of the energy of the incident core particles in both cases ELM or disruption events, despite the large difference in the initial energy released. The quickly developed secondary divertor plasma effectively shields the divertor plate from the direct impact of the hydrogen plasma during the early stages of the disruption event. The ELM is a regular event (~ 1 Hz)^16^ during ITER normal operation and when the majority of the total ELM energy is contained in the highly radiative evolving tungsten denser plasma, serious damage to other nearby and hidden components could occur as well as adverse effects due to potential core plasma contamination that could terminate each ELM into a disruption.

The detailed photon radiation transport calculations, which consumes most of the simulation time, predicted very effective conversion of the core escaped particles kinetic energy into photon radiation from the tungsten generated plasma. More than half of the total incident energy will be converted into photons during an ELM and about that during a disruption. This means that the classical MHD equations used without the secondary plasma photon transport used in most tokamak models ignore the optical properties and energy transfer of half of the initial energy of transient events. One should also note again that photon transport is independent of the complex structure of the tokamak magnetic field and photons are free to go anywhere inside the chamber within line of sight.

While the photon transport is not affected by a magnetic field, the secondary tungsten plasma is and changes/sweeps the initial magnetic field configuration, and therefore changes the photon transport characteristics. As a result, the original impact energy can be re-deposited in unforeseen and hidden interior tokamak surfaces depending on the hydrodynamic evolution of the generated secondary plasma and its photon transport. HEIGHTS full three dimensional simulation of the exact ITER design of the internal components determines where such photons radiation energy deposit their energy and what damage, e.g., melting and vaporization occurs when and where to various internal components during transients.

## Results and discussion

**Figure 3:**
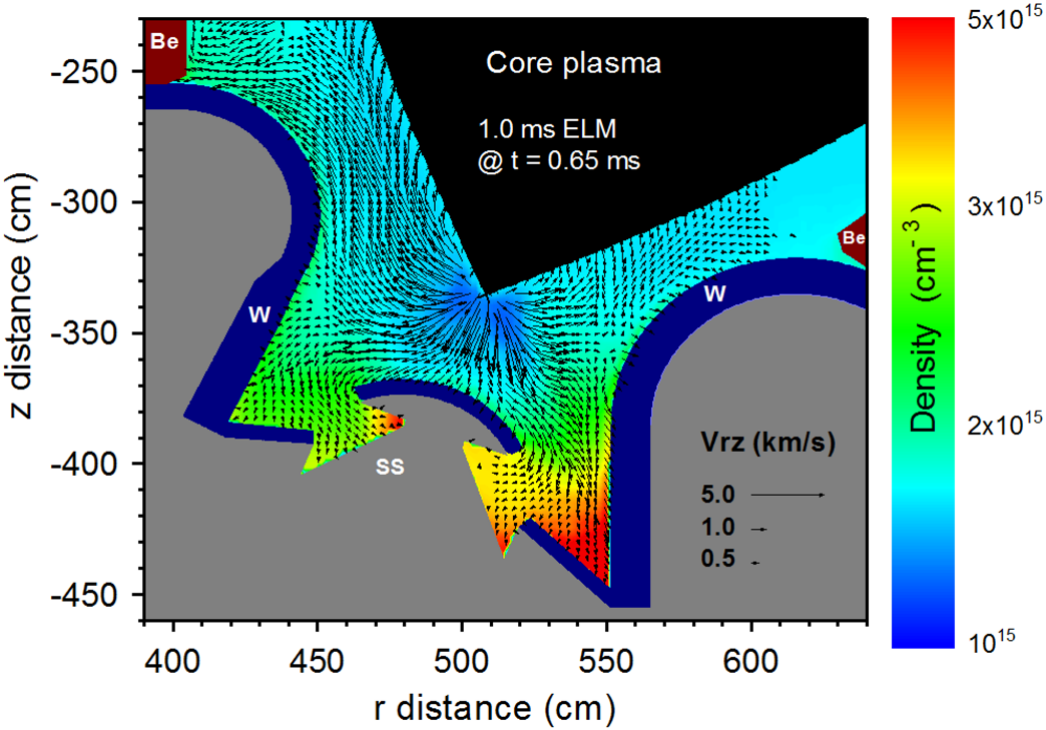
Secondary plasma density and velocity evolution at t = 0.65 ms during the 1.0 ms ELM. The images were prepared using OriginPro V2020.

The integration of all physical processes including detailed photon radiation transport allows self-consistent and accurate balance of the energy transfer to various tokamak surfaces therefore, accurate assessment of the damage incurred^6^. The results of the integrated photon transport and collisional processes in full 3D real ITER geometry with all details of magnetic and electric fields show unexpected and significant heat loads and damage to sensitive components other than the expected damage to the original divertor plate and even including the first walls. In addition, the expansion of the high-Z tungsten plasma towards the core plasma during ELMs which occur at normal operation could terminate the discharge via a disruption; a significant concern. HEIGHTS integrated simulation can predict at any point inside the entire ITER 3D geometry all incident particles (charged particles and photons) and then calculate the energy fluxes (particles deposition and photon radiation) from both the escaped core plasma and from the re-radiated photons emitted from the secondary plasma. Figure 3 shows a snapshot of the secondary W plasma evolution (at t = 0.65 ms) during the 1.0 ms ELM in ITER machine. The tungsten plasma (initiated at the divertor plates) expands into the SOL and penetrates into the core plasma. The ELM is not yet terminated and the escaped core fuel plasma still interacts with this evolving tungsten plasma and cannot be stopped by the low density core plasma. The fuel plasma particles will be collided and scattered by the heavy tungsten particles and cross the magnetic field line as illustrated in Fig. 2.

**Figure 4:**
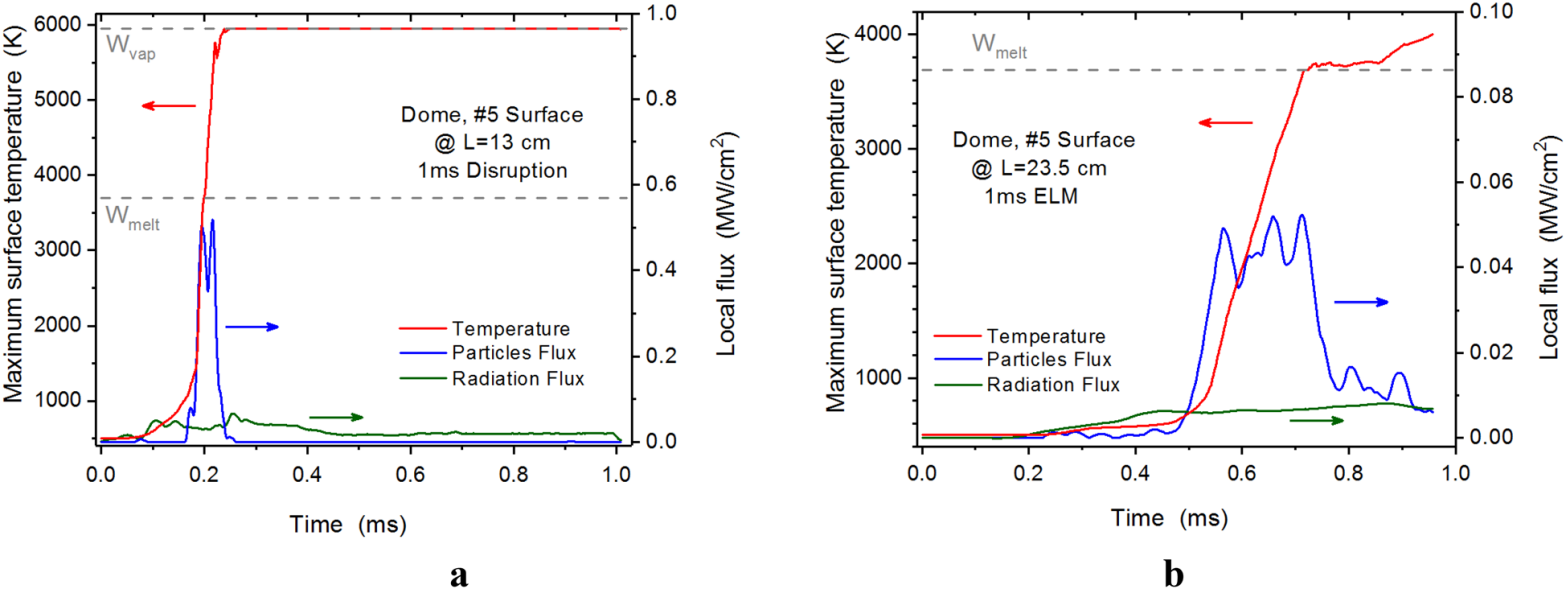
Hot spots on the Dome surface during a disruption (**a**), and during an ELM (**b**). The images were prepared using OriginPro V2020.

As an example of the consequences of the secondary plasma generation, evolution, and redistribution of the transient event energy is the damage occurring on the Dome surface shown in Fig. 4. Initially the Dome surface is not expected to have any significant energy deposition during transient events due to the magnetic configuration. The reradiated energy of the tungsten plasma and the scattered fuel plasma particles energy deposition can result in serious hot spots on the Dome surface leading to significant melting and vaporization (Fig. 4), unpredicted or expected by ITER team without such detail modeling and simulation.

**Figure 5:**
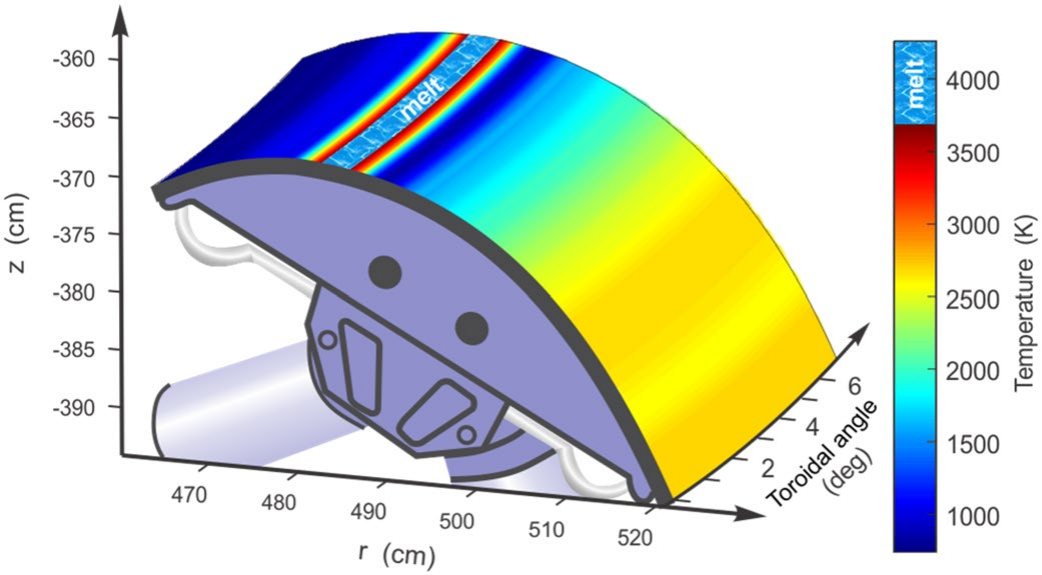
Thermal response of the ITER Dome surface after the 1.0 ms ELM. The images were prepared using OriginPro V2020 and CorelDRAW Graphics Suite 11.

The hot spots or the overheated areas appear in time when the scattered fuel plasma particles combined with photonic radiation flux exceed the thermal damage limit of the material. In the disruption case, the hot spot is localized at surface distance of 13 cm and time t = 0.2 ms (Fig. 4a) while in the ELM case the overheating occurs later at t = 0.8 ms at a surface distance of 23.5 cm (Fig. 4b). The disruption energy is about ten time larger than ELM and Dome surface is even vaporized at that location. Figure 5 shows the surface temperature of the entire Dome surface after the 1.0 ms ELM event and the resulting melted areas.

Despite that the ELMs energy during normal operation is much lower than the rare but serious disruption events, the vaporization of the divertor plate and the subsequent generation of a secondary tungsten plasma soon after the start of the ELM event will have similar consequences as the disruption events in damaging sensitive nearby components. The generated secondary divertor plasma during an ELM is still dense and hot enough to generate substantial photon radiation fluxes. Despite that the radiation fluxes and W plasma density during the ELM event are much smaller in comparison to a disruption, the simulations showed melting spots on the Dome surface up to ~ 15.0 µm in depth (A.H. & V.S. *Nucl. Fusion,* manuscript submitted). The more serious concern in case of ELMs is the penetration of the secondary plasma into the core plasma (Fig. 3) which could result in a disruption during every ELM event.

**Figure 6:**
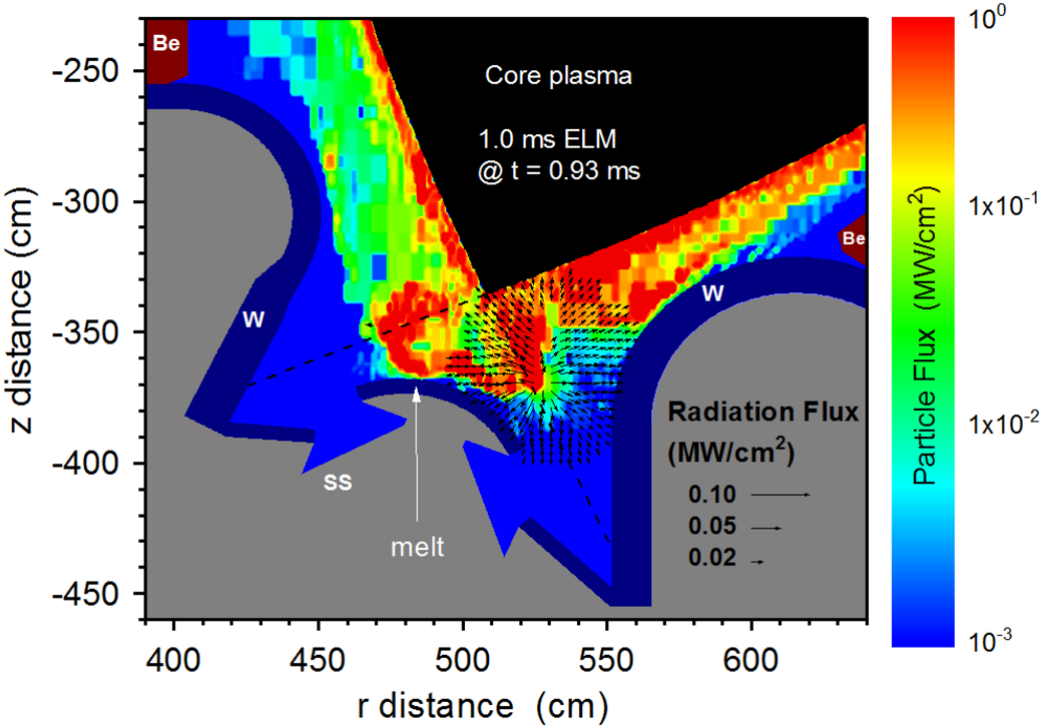
HEIGHTS simulation of particle fluxes (color field) and secondary photon radiation (vectors) in ITER divertor space at t = 0.65 ms of the 1.0 ms ELM. The images were prepared using OriginPro V2020.

The abrupt heating of the Dome surface occurred as a result of the scattered particle showers of the incident core plasma from the front of the dynamically evolving dense secondary plasma (see Fig. 3). The resulting damage is enhanced by the continuous photon radiation of the secondary plasma at these locations as well. The combination of the particles and photon radiation fluxes is shown in Fig. 6.

The divertor and the reflector plates surfaces are expected to have surface damage due to melting and vaporization from the high heat loads during transients. It is believed for such tokamak design and in current ITER as well that simple replacement of these plates every now and then will keep the reactor in good working condition. However, the expected generation of dense divertor plasma, even during ELMs, and its potential penetration and interaction with the core plasma may have serious damage effects and more disruption events.

## Issues with ITER divertor design

The integrated simulations of the real ITER reactor design along with implementing the correct physics show that when increasing the power, compared to current small low-power tokamaks and during transient events, a dense secondary plasma is generated from the divertor plate that will determine the fate and lifetime of all interior components. Even during normal operation in future ITER-like devices will be subjected to ELMs plasma fluxes high enough to cause intense vaporization at the strike points. This secondary generated high-Z plasma will require comprehensive and complex simulations and the current scaling estimations developed for small tokamaks will not be valid. The magnetohydrodynamics, optical plasma properties, and photon transport in such dense plasma are very complicated processes and require careful simulation and proper physics. The HEIGHTS package is well benchmarked over the past decades in these areas for laser and discharge produced plasma applications^11,21^.

There are several concerns in the current tokamak divertor designs. First, the initial incident flux during transients is concentrated in a very narrow area on the divertor plate (around the strike point). Second, the high-Z divertor plates are located not far from the D/T plasma core. Third, the design of the divertor/geometry is not optimized for a highly radiative secondary plasma generation and evolution following transient events.

The focused ELM and disruption fluxes on a very narrow tungsten surface area will generate a highly radiative secondary plasma that transfer the expected transient heat loads initially on the divertor plate to other nearby and hidden components. The belief of simply repairing or replacing the divertor plate when sufficient damage has occurred is not sufficient solution. Depositing large amount of energy in a small and closed area may protect the original divertor plate due to vapor/plasma shielding effects but the intense radiation generated and the scattered plasma particle fluxes from such dense plasma cloud will cause damage to other internal and hidden components in this closed space. In fact, one can predict during transient events in ITER current design that a low-Z divertor plate could protect the sensitive nearby components better than the high-Z tungsten divertor plate despite the more potential repair required for a low-Z divertor plate because of the low radiation power of low-Z materials, e.g., carbon^22^. This could be potential temporary solution to current ITER design, however further simulations are required before such conclusion is recommended.

Other tokamak designs concepts may offer a better solution or better protection methods to this problem such as Snow-Flake and Super-X magnetic configurations^23,24^. Such magnetic configurations and designs allow to distribute the incident core plasma particles over a wider area on the divertor surface and therefore, decrease the heat loads on the divertor plate. Another possible solution for the divertor design is to move the divertor plates and strike points farther from the core plasma^4,25^. Designing a special divertor chamber as described in Ref.^26^ could greatly improve this situation and could limit the potential high-Z drift and penetration into the operating chamber and prevent core plasma contamination.

The low-Z impurity seeding and divertor plasma detachment is considered as a robust solution of the divertor erosion problem particularly in ITER-like devices compared to current tokamak devices. Also, the dense seeding can cause core plasma contamination and the low-density seeding may not be effective due to the large core plasma particle flux. We are also planning for our next integrated simulations assuming the presence of low-density divertor seeding. Mitigation methods have its own uncertainties and not always will work properly. Therefore, in this paper we studied the effects of very small number (even one) of unmitigated disruptions and giant ELMs. Any credible design must tolerate few unmitigated transient events. In our simulation, we assumed the standard prediction of the ELM frequency during the normal operation of current ITER design^14^. There is a high risk of disrupting the plasma every time there is an ELM due to core contamination of the eroded materials.

## Conclusion

A serious concern to any successful concept for energy production in magnetic fusion reactors is their performance during abnormal events including plasma disruptions due to loss of plasma confinement and edge-localized modes (ELMs) during normal operation. A reliable and a successful reactor design should stand several of these transient events without any serious consequences. Our comprehensive HEIGHTS simulation package has unique capabilities including all required physics for simulating in full three dimension any tokamak exact geometry and carefully evaluating components response during transient events and predicting potential damage mechanisms to every internal and hidden component of all divertor cassette structures and the first walls as well. The expected increase in future tokamak power compared to current machines, e.g., ITER and beyond, will increase the power flux on the divertor plates during transient events to cause surface melting, vaporization, and the development of a secondary plasma from the original divertor materials. The secondary plasma will evolve above the divertor surface, expanding along magnetic field lines into scrape-off-layer (SOL), and intercepting the remaining incoming transient disruption or ELM particles, and in case of ELMs could potentially penetrating into the core plasma and possible termination of the discharge into a full disruption following each ELM. The transient events plasma particles energy is mainly converted into two significant secondary heat sources, i.e., photons radiation and scattered particle fluxes that could cause severe erosion and damage to many internal and hidden components that were not exposed directly to disruptions and ELMs. To mitigate the effects of the highly radiative secondary plasma one need to increase the initial core plasma impact area along the divertor plate, to place the divertor plates far away from the tokamak core, or to divert disrupting and ELM plasma into a separate larger compartment. These options will require significant design changes. The performance and optimization of future divertor designs should take into account the secondary divertor plasma evolution, magnetohydrodynamics, expansion into and the diffusion of the magnetic field structure, detail plasma opacity, and accurate photon generation and its radiation transport. The current ITER divertor design will not work properly during transient plasma events and needs to be modified or a new design should be developed to ensure successful operation and maintain the confidence in the tokamak concept as a viable magnetic fusion energy production system.

## Methods

### Simulations setup

HEIGHTS simulation package is developed over the last three decades to study the effects of any form of intense energy or power deposition on target materials and components for various energy, industrial, and defense applications. We have extensively upgraded HEIGHTS to study and simulate the effects of transient plasma events in magnetic fusion devices such as ITER and future DEMO machines. The initial simulation conditions for ITER device included three main parameters: (1) pedestal energy *W*_*ped*_ = 126 MJ, (2) temperature of pedestal *T*_*ped*_ = 3.5 keV, and (3) transient event duration *τ* = 0.1 − 3.0 ms to cover all possible transient duration range. We used a total of 126 MJ for disruption and 12.6 MJ for giant ELM. We varied the event duration in the expected range of 0.1–3.0 ms but in this study we only focused on the most anticipated duration of 1.0 ms. The initial conditions of the equilibrium magnetic plasma configuration used in our study was obtained from the EQDSK database files^10^. We simulated the initiated divertor plasma evolution and its atomic physics, opacity, and radiation characteristics during and after an ELM or a disruption in ITER current exact three dimensional design^27^. Details of the ITER design implementation on an adaptive mesh are described in Ref.^6^.

The Bebop cluster at the Argonne National Laboratory^28^ was used for our simulations.

### Magnetohydrodynamics of the secondary plasma

The integrated models and packages were constructed in the 3D coordinate system where the secondary plasma MHD equations set is used as the model core^6,8^. The equations system describes the convective motion of the secondary plasma and should be coupled with the dissipative physical processes such as the heat conduction, radiation transport, magnetic diffusion, and the core plasma energy deposition during the transient events. Since the common equations combine all physical processes as convective (hydrodynamic flux) and dissipative (heat conduction, radiation transport, disruption plasma impact), we used splitting of the physical processes in our numerical algorithm to separate the hyperbolic and parabolic parts^29,30^. The convective part was reduced to matrix form and solved following the total variation diminishing scheme in the Lax–Friedrich formulation (TVD-LF)^31^.

### Magnetic diffusion and plasma heat conduction

The magnetic diffusion equations have parabolic type and for its solution we used an implicit method based on sparse matrix solution. The solution determines dissipative changes of magnetic field that should be used for the correction of the MHD. The details of magnetic diffusion and plasma heat conduction in the evolving secondary plasma were extensively developed in Ref.^30^. We consider the dense secondary plasma here assuming transport coefficients studied in Refs.^32–35^.

### Kinetic Monte Carlo simulation of escaping core plasma particles

The model of core plasma particles evolution includes eight main scattering processes: ion–nuclear interactions, ion–electron interaction, electron–nuclear interaction, electron–electron interaction, Bremsstrahlung process, Compton processes, photoabsorption, and Auger recombination. The approximation of binary or pair collisions^36^ is used for simulations. A detailed description of the core-plasma-escaping models and its numerical implementation with initial and boundary conditions are presented in Refs.^6,8^.

### Radiation transport and opacities of secondary plasma

Our recent integrated model^6^, was enhanced to allow the escaped core particles deposit part of their energy in the evolved vapor and then the secondary plasma during the collisions. For detail calculation of the secondary radiation, we developed a comprehensive Monte Carlo radiation transport (RT) model based on several weighted factors to significantly reduce the computational time^17,37^. The implemented weight factors in our MC model greatly reduced RT calculations compared to both MC models without weight factors and the direct RT solution methods^18^. The details of the MC RT methods executed for the quadtree mesh were published elsewhere^6,8^.

In calculating the radiation transport in plasma, the integral radiation fluxes depend to a great extent on the level of detail and the precision of the optical coefficients^38^. The atomic characteristics are calculated using self-consistent Hartree–Fock–Slater method^39^. The collisional radiation equilibrium (CRE) model was used to calculate the populations of atomic levels and the ion and electron plasma concentrations^40^. The escape probability approximation for line transitions and direct photoionization for the continuum spectrum was implemented to reduce the nonlocal radiation effects^41^. Because details of opacity and atomic data calculations are very extensive, we refer to our previous publications^38,42^.

## Data Availability

The data that support the findings of this study are stored on Purdue Servers and on Argonne National Laboratory Bebop cluster and are available from the corresponding authors upon reasonable request.
